# Complete genome sequences of five *Variovorax* strains isolated from the *Populus* rhizosphere and endosphere

**DOI:** 10.1128/mra.00990-25

**Published:** 2025-12-08

**Authors:** Delaney G. Beals, Alyssa A. Carrell, Brisa Rodriguez, William G. Alexander, Christopher W. Schadt, Leah H. Hochanadel, Mircea Podar, Dale A. Pelletier

**Affiliations:** 1 Biosciences Division, Oak Ridge National Laboratory 6146, Oak Ridge, Tennessee, USA; Indiana University, Bloomington, Bloomington, Indiana, USA

**Keywords:** plant-microbe interface, endosphere, rhizosphere, poplar, Variovorax

## Abstract

We report the complete genome sequences of five *Variovorax* strains isolated from the roots of *Populus* species in greenhouse and field settings. Genome sizes range from 7.44 to 7.71 Mbp and encode 6,077–6,879 predicted protein-coding genes. These genome assemblies expand the known genomic catalog of *Variovorax* in plant-associated environments.

## ANNOUNCEMENT

The bacterial genus *Variovorax* (Burkholderiales, *Comamonadaceae*) comprises metabolically versatile bacteria inhabiting soils and plant roots (1–6). We report complete genome sequences of strains BK018, GV025, GV048, YR566, and WT075, isolated from *Populus*-associated habitats to support ecological and functional diversity studies within this genus.

Strains were isolated from *Populus* root-associated compartments using dilution plating on R2A agar incubated at 28°C for 2 days. BK018 originated from a field-grown *P. deltoides* tree in Bellville, GA (32.1348° N, 81.9657° W), and YR566 from a *P. deltoides* tree in Jerusalem, NC (35.8381° N, 80.4856° W). WT075 was recovered from the rhizosphere soil of a *P. trichocarpa* tree in Naches, WA (46.7024° N, 120.6601° W). GV025 and GV048 were obtained from surface-sterilized roots of *P. trichocarpa* plants grown in a greenhouse (Oak Ridge National Laboratory), inoculated with soil collected from a *P. trichocarpa* common garden in Corvallis, OR (44.5880° N, 123.1935° W). Taxonomic identification preceded genome sequencing by 16S rRNA gene amplification (primers 27F/1492R), Sanger sequencing (Eurofins Genomics, Louisville, KY, USA), and BLASTn comparison against the NCBI rRNA database, showing ≥99% identity to *Variovorax* species.

Frozen stocks were revived on R2A agar, and single colonies were grown in R2A broth at 28°C overnight for DNA extraction. Genomic DNA was isolated using the DNeasy Blood and Tissue Kit (Qiagen) and eluted in 0.1× TE buffer. Libraries for WT075 were prepared in-house using the Oxford Nanopore Technologies (ONT) Ligation Sequencing Kit without shearing or size selection and sequenced on an R10.4.1 MinION flow cell. Libraries for the remaining strains were prepared by Plasmidsaurus (Louisville, KY, USA) using the ONT Ligation Sequencing Kit and sequenced on GridION R10.4.1 flow cells with V14 chemistry. High molecular weight DNA was tagmented without additional shearing or size selection, following the amplification-free ligation protocol. Base calling for WT075 used Dorado v0.7.1 (ONT) with the high-accuracy calling model; for the remaining strains, Guppy v6.4.6 (ONT) was used. Each strain yielded 87k–104k reads (N50: 15.2–18.6 kb). Reads filtered with Filtlong v0.2.1 (minimum Q ≥ 10, top 95% retained) were assembled with Trycycler v0.5.4 (7) using Flye v2.9.4 (8), Raven v1.5.3 (9), and Miniasm v0.3 (10, 11) and polished with Minipolish v0.1.3 (12) and Medaka v2.0.1 (ONT). Default parameters were used unless noted.

WT075 was assembled into a single circular chromosome, while all others each yielded two circular contigs. Sequencing coverage ranged from 40.7× to 72.0× (Table 1). Genome sizes ranged from 7.44 to 7.71 Mbp with GC contents between 66.1% and 69.3%. Prokka v1.14.6 (13) predicted 6,077–6,879 protein-coding genes per genome (14). Circularity was confirmed by identifying overlapping ends and trimming with Trycycler; contigs were not rotated to a specific gene.

**TABLE 1:** Genome statistics for newly sequenced *Variovorax* strains

Strain	Genome size (bp)	GC (%)	N50	Predicted genes	Completeness (%)/contamination (%)	Contig 1 length (bp)	Contig 1 coverage
						Contig 2 length (bp)	Contig 2 coverage
BK018	7,593,393	67.7	6,331,524	6,618 (6,611 unique)	100/0.47	6,331,524	65.6
						1,261,869	63.8
GV025	7,489,868	69.3	5,711,485	6,085 (6,077 unique)	100/0.53	5,711,485	62.8
						1,778,383	72
GV048	7,489,859	69.3	5,711,476	6,085 (6,078 unique)	100/0.53	5,711,476	66.5
						1,778,383	66.3
WT075	7,447,132	67.1	7,447,132	6,517 (6,470 unique)	100/0.62	7,447,132	40.7
	
YR566	7,705,554	66.1	7,249,798	6,879 (6,852 unique)	100/0.47	7,249,798	65.1
						455,756	56.5

**Fig 1:**
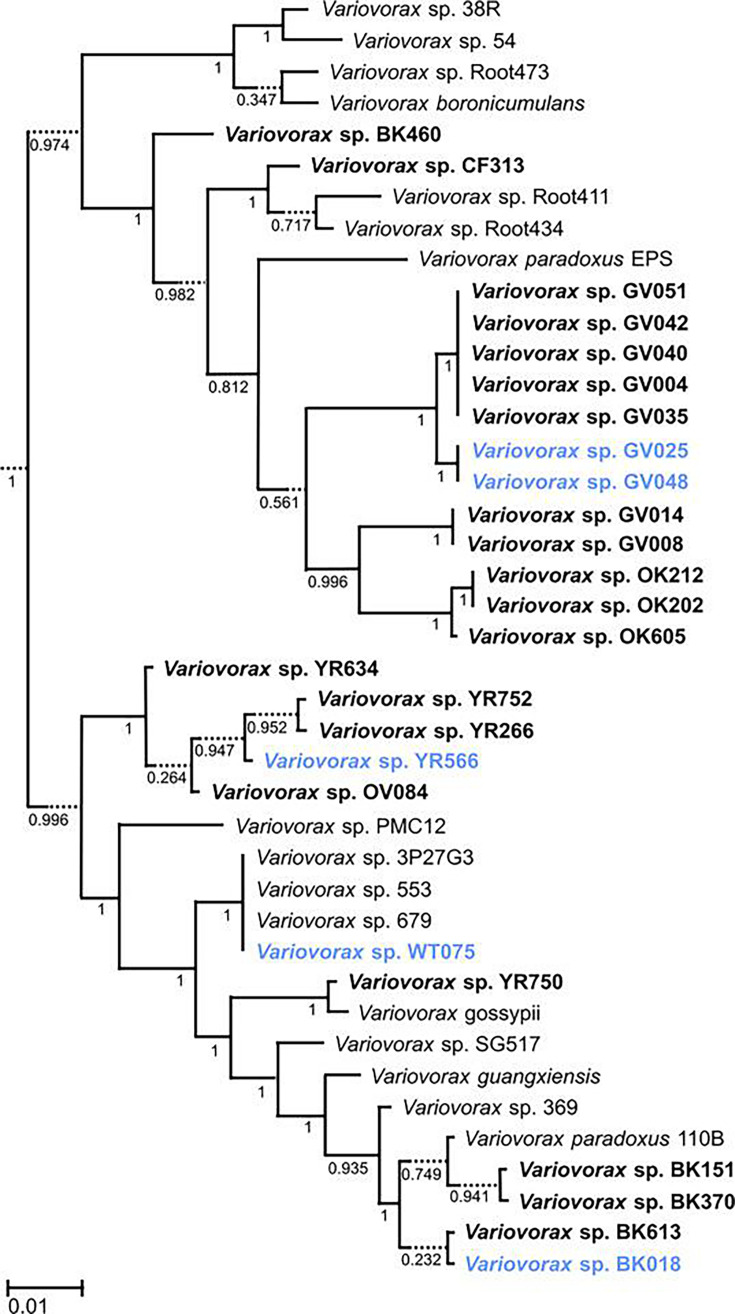
Maximum-likelihood phylogenetic tree based on 49 core universal bacterial proteins computed using SpeciesTree v2.2.0 in KBase. The five newly sequenced strains are shown in blue. *Variovorax* spp. strains previously isolated from *Populus* trees are shown in bold. Branch support values and scale bar indicate substitutions per site.

Taxonomic classification (GTDB-Tk v2.3.2 [(15]), ANI comparisons (FastANI v0.1.3 [16]), and phylogenetic placement (SpeciesTree v2.2.0 [17], Fig. 1) were performed in KBase (18). BK018 was identified as *Variovorax paradoxus* A and WT075 as *Variovorax* sp003952185 (ANI ≥98.6%). GV025 and GV048 shared 99.99% ANI and grouped with *Variovorax* sp. OK212 (91.6% ANI), while YR566 was classified as *Variovorax* sp900106655 (95.8% ANI).

## Data Availability

Unprocessed sequencing reads have been deposited in GenBank under BioProject accession number PRJNA1255178. Individual genome assemblies have been deposited to GenBank under the accession numbers CP197088–CP197089, CP197086–CP197087, CP197084–CP197085, CP197083, and CP197092–CP197093. A KBase Narrative containing all sequence data and genome analysis steps is also publicly accessible (14).
